# Augmented efficacy of exogenous extracellular vesicles targeted to injured kidneys

**DOI:** 10.1038/s41392-020-00304-6

**Published:** 2020-09-14

**Authors:** Xiao-Jun Chen, Kai Jiang, Christopher M. Ferguson, Hui Tang, Xiangyang Zhu, Amir Lerman, Lilach O. Lerman

**Affiliations:** 1 Division of Nephrology and Hypertension, Mayo Clinic, Rochester, MN USA; 2 Department of Nephrology, The Second Xiangya Hospital of Central South University, Changsha, Hunan China; 3 Department of Cardiovascular Diseases, Mayo Clinic, Rochester, MN USA

**Keywords:** Cell biology, Kidney diseases

**Dear Editor**,

Extracellular vesicles (EVs) derived from mesenchymal stem/stromal cells (MSCs) contain genetic and protein material that stimulate tissue repair and ameliorate injury in recipient cells. Advantages of particulate MSC-EVs over MSCs in treating kidney disease include better penetration of injured glomerular filtration barrier to access podocytes or tubular cells. However, systemic EV delivery yields low kidney retention efficiency, limiting their regenerative benefits.^1^ Previously, we coated adipose tissue-derived (AD)-MSC with antibodies against kidney injury molecule (KIM)‐1 (ab-KIM1), a protein upregulated in damaged kidneys.^2^ Conjugating ab-KIM1 did not impair MSC function but increased their retention and reparative potency in murine renal artery stenosis (RAS).^2^ We hypothesized that ab-KIM1 conjugation would similarly enhance retention of exogenously delivered EVs in ischemic kidneys and confer superior therapeutic benefits.

We collected from abdominal murine AD-MSCs supernatant 100–180 nm EVs (Supplementary Fig. 1a). Using palmitated protein G bridge, we coated EVs, pre-stained with fluorescent membrane-dye (DiO green), with allophycocyanin (APC)‐conjugated ab-KIM1 (Supplementary Materials), achieving 90% coating efficiency (APC+/DiO+, Fig. 1a).

**Fig. 1:**
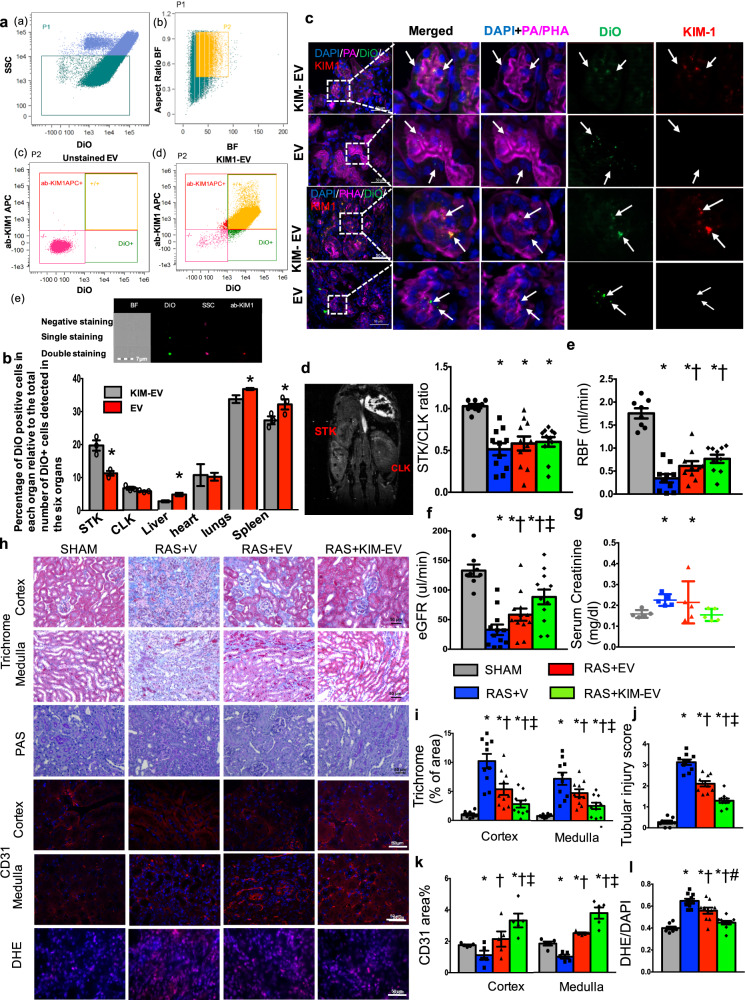
**a** Flow gating strategy: **a** side-scatter [SSC](low) population (P1), events <1 µm, were gated from total events; **b** P1 population was gated to exclude doublets (P2); **c**, **d** SSC(low) DiO(+) events were considered EVs. Allophycocyanin [APC](−)DiO(−) and APC(+)DiO(+) populations were sorted from P2, with 99% unstained extracellular vesicles (EVs) APC(−)DiO(−) and 90% DiO(+) EV coated with ab-KIM1; **e** Images of ab-KIM1-coated EV (scale bar = 7 μm) double positive for allophycocyanin (red) and Dio (green). BF bright field, ab‐KIM1 antibodies against kidney injury molecule‐1. **b** Percentages of cells engrafting EVs detected in the heart, lungs, liver, spleen, and kidneys by imaging flow cytometry. *p < 0.05 vs. native-EV. **c** Immunofluorescent co-staining with *Phaseolus vulgaris* erythroagglutinin (PHA-E) and peanut agglutinin (PA) (magenta) identified EV fragments (green, DiO) within stenotic kidney (STK) proximal and distal tubular cells. **d** STK/contralateral kidney (CLK) volume ratio decreased similarly in all the RAS groups. **e** STK-RBF decreased in RAS, increased similarly by native-EVs and KIM-EV, but remained lower than Sham. **f** STK-GFR decreased in RAS and improved only by KIM‐EV. **g** Higher plasma creatinine in RAS vs. Sham was unaffected by EVs but normalized by KIM-EVs. **h** STK cortex and medulla staining of trichrome (×20), PAS (×20), CD31 (red, ×40), and dihydroethidium (DHE) [×40, pink, nuclei blue] after treatment with Vehicle (V), EV, or KIM‐EV. **i**, **j** Renal fibrosis and tubular injury increased in RAS + Vehicle. KIM‐EV attenuated them further than RAS + EV. **k** STK CD31 staining showed a decrease in microvascular density, which improved with EV, and significantly more by KIM‐EV. **l** Elevated STK-DHE indicated increased superoxide production, which decreased by EV and tended to decrease further by KIM‐EV. *p < 0.05 vs. Sham, ^†^*p* < 0.05 vs. RAS + vehicle, ^‡^*p* < 0.05 vs. RAS + EV, ^#^*p* = 0.1 vs. RAS + EV

Then untreated AD-MSC-EV, ab-KIM1-coated-EV (KIM-EV), or vehicle were injected into murine aorta after 2 weeks of unilateral RAS^2^ (Supplementary Materials). Two weeks later, mice underwent in vivo micro-magnetic resonance imaging (micro-MRI) studies of single kidney function, followed by ex vivo studies. Flow cytometry (Fig. 1b) and histology (Supplementary Fig. 1b) revealed, compared to native-EVs, increased DiO+ cells in the stenotic kidneys (STKs) (19.7 ± 2.7 vs. 11.3 ± 1.0%, *p* = 0.01), slight elevation in the contralateral kidney (CLK) (6.6 ± 0.8% vs. 5.7 ± 0.2%, *p* = 0.07), modest decrements in the spleen, liver, and lungs (*p* < 0.05), and unchanged in the heart. EV retention rate (CD63+/DiO+ area) confirmed greater KIM-EV vs. native-EV retention (Supplementary Fig. 1c).

To demonstrate EV binding to KIM-1, HK2 cells were injured with increasing cisplatin concentrations (0–25 µM) for 24 h. Additional cisplatin-pretreated (20 µM) HK2 were incubated with EVs for 1 h. KIM-1 expression rose progressively with cisplatin concentrations (Supplementary Fig. 2a), and bound more KIM-EVs than native-EVs (Supplementary Fig. 2b). In vivo, EV homing, retention, and uptake in STKs and tubular cells increased compared to non-injured organs, possibly secondary to adhesion molecules, and KIM-1 conjugation increased this further. Co-localization with distal (peanut agglutinin) and proximal (*Phaseolus vulgaris* erythroagglutinin) tubular markers confirmed engraftment (Fig. 1c).

Micro-MRI demonstrated comparable stenosis (decreased STK/CLK volume ratios) in the RAS groups (Fig. 1d). STK-RBF, which decreased in RAS, improved similarly by native-EV and KIM-EV (Fig. 1e), but STK-GFR was improved only by KIM‐EV (Fig. 1f), as were elevated creatinine levels (Fig. 1g). Hence, KIM-EVs were more effective than native-EVs in restoring renal function. Contrarily, blood pressure (tail-cuff) remained unchanged (Supplementary Fig. 2c), and both EV types blunted medullary hypoxia (R2*) (Supplementary Fig. 2d).

Congruently, KIM-EV-treated mice manifested greater attenuation of STK tubular injury, capillary loss, oxidative stress, and fibrosis (Fig. 1h–l). Tubular cells are susceptible to injury, and inadequately repaired cells may prompt fibrosis and inflammation. While AD-MSC-EV blunt STK tubular injury and fibrosis,^3^ ab-KIM-1 conjugation enhanced their STK retention. Double staining indicated that intact KIM-EV engrafted in tubules and might have possibly conferred resistance to injury and improved STK-GFR.

Remodeling and loss of microcirculation also mediate renal ischemic disease progression, yet MSC-derived EVs are endowed with pro-angiogenic properties. In this study, EV delivery improved peritubular capillary density. The robust pro-angiogenic effects of KIM-EV might stem from repair of tubular cells that regulate microvascular development^4^ and reduced oxidative stress and fibrosis.^2^ Furthermore, while both decreased the upregulated STK gene expression of *VEGF* and *Flk-1* (likely compensatory to ischemia), only KIM-EV normalized *Angpt-1* expression (Supplementary Fig. 1e). Angiopoietin-1 is expressed in renal cortex epithelia and upregulated by ischemia and angiotensin-II.^5^ Angiopoietin-1 can blunt angiogenesis when pro-angiogenic factors are upregulated and promote fibrosis and inflammation.^5^ Therefore, augmented KIM-EV tubular engraftment might have downregulated *Angpt-1 and Mcp-1* and, in turn, STK oxidative stress, inflammation, capillary loss, and fibrosis.

Interestingly, EVs and KIM-EV both attenuated pro-inflammatory gene expression, including *intercellular cell-adhesion molecule-1*, *interleukin-6*, and *tumor necrosis-factor-α* (Supplementary Fig. 2e). However, KIM-EVs were more effective in downregulating *monocyte chemotactic-protein-1*, a mediator of ischemic injury in renal tubules, suggesting a greater anti-inflammatory potency of KIM-EV and efficacy to attenuate kidney damage.

Therefore, we introduce a novel approach to target MSC-derived EVs to injured kidneys. EV coating with ab-KIM1, a specific marker of kidney injury, increased their retention in the ischemic kidney and enhanced their therapeutic effects. Our study extends strategies for EV-based treatment in ischemic kidney injury, as well as their broad applications using comparable strategies.

## Supplementary information

Supplementary Figure 1

Supplementary Figure 2

Supplementary Text

## Data Availability

All data relevant to this work are included in this paper and Supplementary Information.
