# Supplementary figures and images for: Augmented efficacy of exogenous extracellular vesicles targeted to injured kidneys
Source: Signal Transduct Target Ther. 2020 Sep 14;5:199. doi: 10.1038/s41392-020-00304-6 (PMC7490711; doi:10.1038/s41392-020-00304-6)

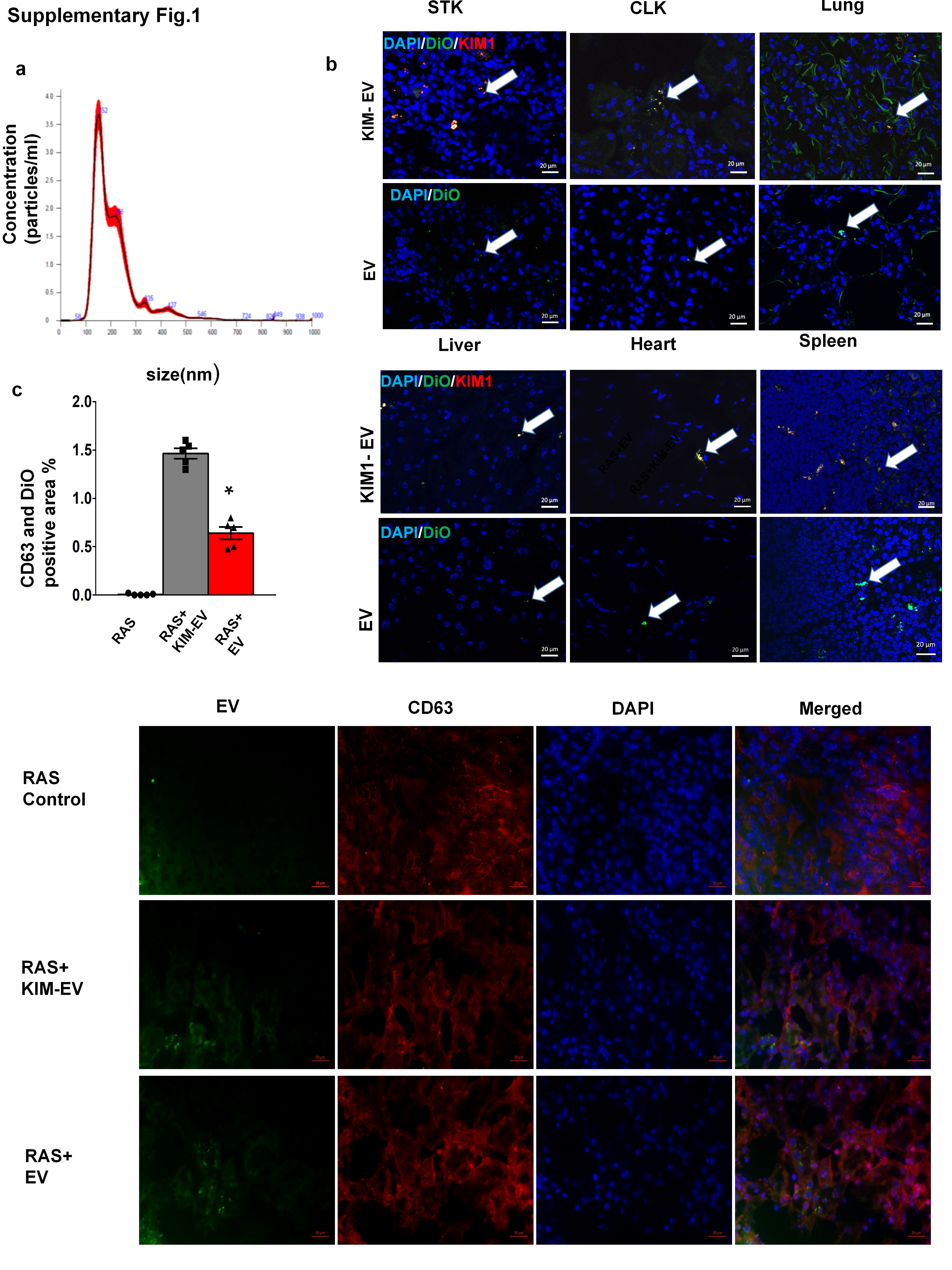

Supplement: Supplementary file 1 — Supplementary Figure 1 [file 41392_2020_304_MOESM1_ESM.jpg]

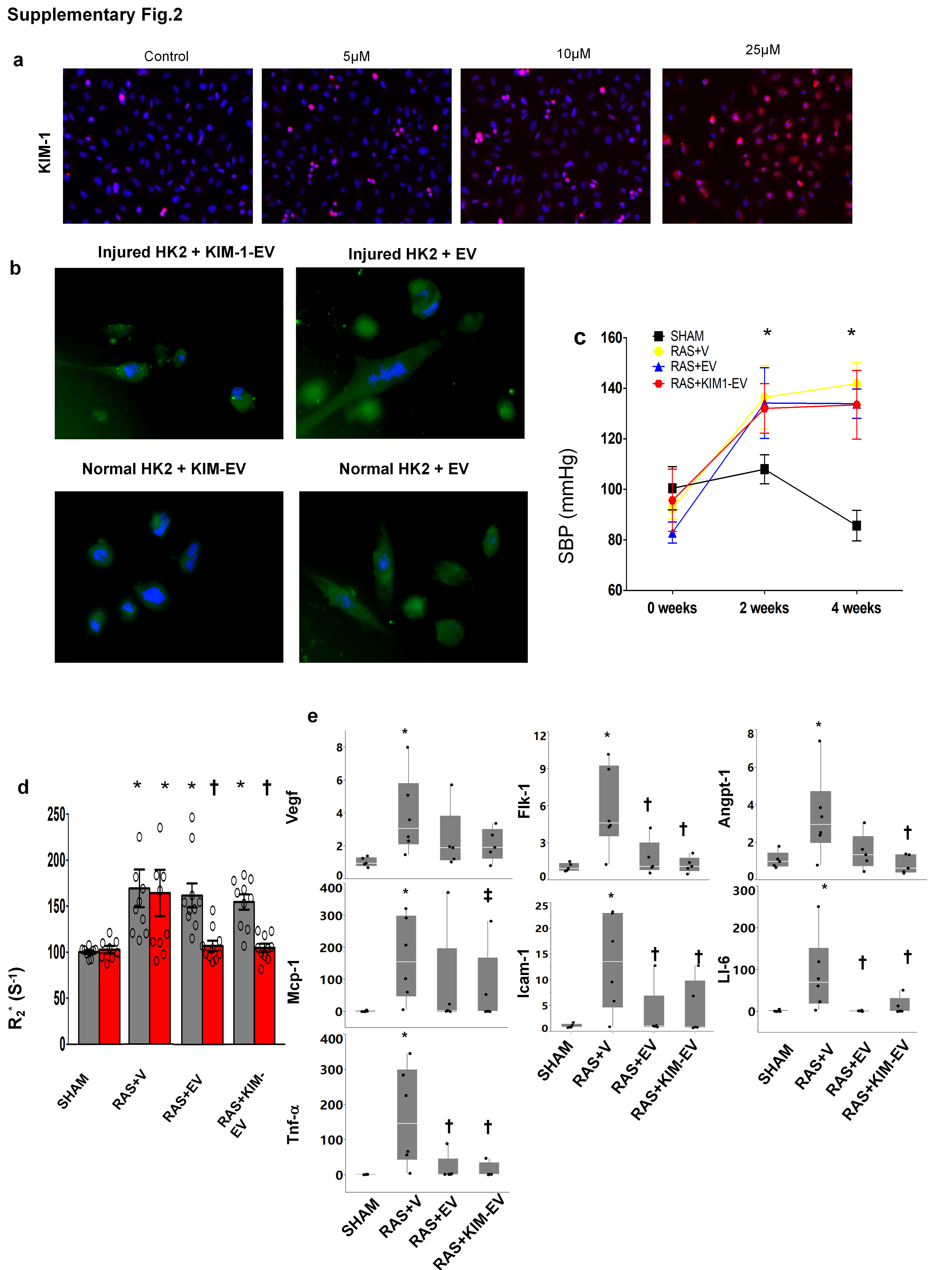

Supplement: Supplementary file 2 — Supplementary Figure 2 [file 41392_2020_304_MOESM2_ESM.jpg]
