# Supplementary material for: Augmented efficacy of exogenous extracellular vesicles targeted to injured kidneys
Source: Signal Transduct Target Ther. 2020 Sep 14;5:199. doi: 10.1038/s41392-020-00304-6 (PMC7490711; doi:10.1038/s41392-020-00304-6)
Supplement: Supplementary file 3 — Supplementary Text [file 41392_2020_304_MOESM3_ESM.docx]

**Augmented efficacy of exogenous extracellular vesicles targeted to injured kidneys**

Xiao-Jun Chen^1, 3^, Kai Jiang^1^, Christopher M. Ferguson^1^, Hui Tang^1^, Xiangyang Zhu^1^, Amir Lerman^2^, Lilach O. Lerman^1, 2^

**Affiliation:**

^1^Division of Nephrology and Hypertension and ^2^Department of Cardiovascular Diseases, Mayo Clinic, Rochester, MN, USA; ^3^Department of Nephrology, The Second Xiangya Hospital of Central-South-University, Changsha, Hunan, China.

**Correspondence:**

Lilach O. Lerman, MD, PhD, Division of Nephrology and Hypertension, Mayo Clinic, 200 First Street SW, Rochester, MN 55905.

Phone:(507)-266-9376 Email:[lerman.lilach@mayo.edu](mailto:lerman.lilach@mayo.edu)

ORCID: 0000-0002-3271-3887

**This PDF file includes:**

Materials and Methods

Supplementary Text

Figures. S1 to S2

**Materials and Methods**

Eleven-week-old 129‐S1 mice (Jackson Lab, Bar Harbor, ME) were studied for 4 weeks after approval by the Mayo Clinic Institutional Animal Care and Use Committee. Mice were randomly divided into sham (n=8), RAS+vehicle (n=10), RAS+EV (n=10), and RAS+KIM‐EV (n=10).

Unilateral renal artery stenosis (RAS) was induced by placing a 0.01-inch diameter PE cuff (BrainTree Scientific) local irritant coil in the right main renal artery, as previously described. Sham surgeries were performed in the control group without placement of a cuff. Blood pressure (BP) was measured at baseline, 2, and 4 weeks after surgery by tail‐cuff (Kent Scientific, Torrington, CT).

Two weeks after surgery, the carotid artery was cannulated via a vascular cut down, and 200µl phosphate buffered solution (PBS), EV, or KIM‐EV (about 1×10^8^ in 200 µL PBS) slowly injected into the aorta. Two weeks later renal function and oxygenation were assessed using a 16.4-T vertical magnetic resonance imaging (MRI) unit (Bruker Biospin, Billerica, MA). Subsequently, the mice were euthanized with CO_2_, and kidneys and blood samples collected for ex-vivo studies. Heart, Lung, spleen and liver were also collected for distribution analysis.

In-Vivo Studies

Two weeks after EVs or vehicle injection, kidney volume and perfusion were measured by non-contrast-enhanced MRI, and single-kidney glomerular filtration rate (GFR) by dynamic contrast-enhanced MRI, as previously described^1^. Renal volume was quantified from images acquired using a respiration‐gated 3D Fast Imaging with Steady Precession Sequence. Renal perfusion was measured using arterial spin labeling with the flow‐sensitive alternating inversion‐recovery sequence, with rapid acquisition with relaxation enhancement images. To measure single-kidney GFR, immediately before an injection of 37.5mM gadodiamide (0.03mmol/kg) through tail-vein over 2s, a fast T1 measurement method saturation recovery snapshot fast low-angle shot (Snapshot-FLASH) was implemented to trace gadolinium dynamics, and a modified two-compartment model used to fit gadolinium dynamics in renal parenchyma. Renal oxygenation was assessed using blood oxygen-level-dependent MRI. T2* was quantified by pixelwise mono‐exponential fitting on the averaged magnitude of all eight images over echo times, and R2* (1/T2*) used as an index of blood oxygenation level.

Ex-Vivo Studies

*MSC and EV Isolation*

MSCs were isolated from abdominal subcutaneous adipose tissue (around 1g) of adult donor mice (129‐S1, 11 weeks) using collagenase, cultured with advanced MEM medium (Gibco/Invitrogen) supplemented with 5% platelet lysate (PLTmax, Mill Creek Life Sciences, Rochester, MN, USA) in 37°/5% CO_2_, and kept in cell recovery medium at –80°C. The third passages of MSCs were characterized by the expression of common MSC markers (CD44, CD90, and CD105) and their potential to differentiate into adipocytes, chondrocytes, and osteocytes was assessed as previously shown^2^.

EVs were isolated from supernatants of MSCs (10×10^6^), cultured in advanced MEM medium without supplements for 48h, using the ultra-centrifugation method^3,4^. In brief, after two initial centrifugations at 2000g and 100,000g (Beckman Coulter Optima L-90 K) for 1h at 4°C, EVs were washed in serum-free medium 199 containing HEPES 25mM, and underwent a final ultra-centrifugation before being stored at –80°C. Concentration and size distribution of isolated EVs was assessed by nanoparticle tracking analysis (NTA) using NanoSight NS300. EVs were diluted with PBS and samples continuously run through a flow-cell top-plate at 50-80μL/min. Ten 30-sec long videos tracking Brownian motion of nanoparticles were recorded and analyzed using NTA 2.3.5^5^.

*EV Coating*

Recombinant protein‐G (RPG, Sigma, MO) was derivatized with N‐hydroxysuccinimide ester of palmitic acid (Sigma, MO) by palmitation reaction as previously described^2^. Sephadex G‐25 column (Sigma, MO) was used to purify the palmitated protein-G (PPG), and protein concentration measured with bicinchoninic acid (BCA) Assay. EVs were suspended at a density of 1×10^10^/ml in serum-free medium-199. PPG was added to the EVs suspension to yield the final concentration at 50 µg/ml and incubated for 1 hour at 37°C. EVs were washed with serum-free medium-199 containing HEPES 25mM, underwent ultra-centrifugation, and then incubated with 100 µg/ml monoclonal rat Allophycocyanin (APC)‐conjugated anti‐KIM1 antibody (R&D Systems) in PBS for another 1 hour at 37°C.

To assess the efficiency and binding rate of the antibody onto the EV surface, isolated EVs were stained for 2 hours at 37°C with 0.5 mmol/L fluorescent membrane dye (DiO Cell Membrane Dye, Molecular Probes) before coating. After coating, the percent of APC (red) and DiO (green) positive EVs was calculated using FlowSight Imaging Flow Cytometer (Amnis Corporation, Seattle, WA) equipped with INSPIRE software as previously describe^6,7^.

*EV Tracking*

Two weeks after delivery, labeled EV were tracked and localized in frozen 5μm sections of the stenotic kidneys by immunofluorescence staining with the distal tubular marker peanut agglutinin (PA, Vector Lab) and the proximal tubular marker Phaseolus vulgaris erythroagglutinin (PHA-E, Vector Lab). Additionally, labeled EVs were tracked in frozen 5 μm sections of the heart, lung, spleen and liver.

*EV Distribution Analysis*

To assess EV bio‐distribution, cells from several organs were analyzed by FACs. Briefly, ice‐cold PBS was used to flush out blood from harvested organs. Similar size sections of fresh STK, contralateral kidney (CLK), heart, lung, spleen and liver were diced and digested with 2 mg/ml collagenase. Digestion was stopped by adding Medium-199 (Gibco BRL) containing 3% FBS. Cells were filtered by passing through a 100µm cell strainer, then centrifuged at 200G for 10 minutes, the pellet re-suspended as single cells, and analyzed by FACS for DiO and DAPI positive cells. The percentage of positive cells in each organ relative to the total number of DiO+ cells detected in the six organs was then calculated. Meanwhile, because EV marker CD63 were used to analyze cellular uptake of EVs in other studies, paraffin kidney slides were stained with CD63, and EV retention rate in kidney was calculated as percentage of double positive area per field (CD63 and DiO positive).

*Renal Injury Evaluation*

Kidney fibrosis was evaluated by Masson's Trichrome staining, and quantified semi-automatically as percent area staining in 5‐µm sections of each kidney (AxioVision, Carl Zeiss Micro Imaging, Thornwood, NY) in 10–15 random fields from each section. Tubular injury (tubular dilation, atrophy, cast formation, sloughing tubular epithelial cells, or thickening of basement membrane) was assessed in sections stained with Periodic acid–Schiff slides on a 0–4 scale (0:<10%, 1:10–25%, 2:26–50%, 3:51–75% and 4:>75% injury). To evaluate microvascular density, kidneys were stained with CD31 antibody (Cell Signaling, 1:100) and a secondary antibody Alexa Fluoro 594 (Santa Cruz, 1:100)^8^. Oxidative stress ex-vivo was evaluated by the in-situ production of superoxide anion (dihydroethidium, DHE). Real-time quantitative PCR was done in kidney tissue using the following Taqman probes (Thermo-Fisher Scientific): *vascular endothelial growth factor* (*Vegf*) (mm00437306)*, fetal liver kinase 1* (*Flk-1*) (mm00440085)*, angiopoietin-1* (*Angpt1*) (mm00456503)*, monocyte chemotactic protein-1* (*Mcp-1*) (mm00441242)*, intercellular cell adhesion molecule-1* (*Icam-1*) (mm00526023)*, interleukin-6* (*IL-6*) (mm00446190)*, and tumor necrosis factor-α* (*Tnf-α*) (mm00443258) , and *Gapdh* (mm99999915) as an internal control.

EV in vitro study

HK2 cells were cultured to demonstrate EVs binding to KIM in vitro. Firstly, HK2 cells were treated with cisplatin with different concenntration for 24h in chamber slides and then fixed them with 4% paraformaldehyde. Then the HK2 cells were incubate with EVs or KIM-EVs (stained with DiO) for 1h.

*Statistical Analysis*

Statistical analysis used the JMP software. Data are expressed as mean±SD or median (range). Statistical significance was assessed by one‐way analysis of variance followed by unpaired two‐tailed t-test for normally distributed data or nonparametric (Wilcoxon and Kruskal‐Wallis) test for no‐normally distributed data. A p≤0.05 was considered significant.

**References**

1 Jiang, K. *et al.* Measurement of Murine Single-Kidney Glomerular Filtration Rate Using Dynamic Contrast-Enhanced MRI. *Magn Reson Med*. **79**, 2935-2943, (2018).

2 Zou, X. *et al.* Targeting Murine Mesenchymal Stem Cells to Kidney Injury Molecule-1 Improves Their Therapeutic Efficacy in Chronic Ischemic Kidney Injury. *Stem Cells Transl Med*. **7**, 394-403, (2018).

3 Conley, S. M. *et al.* Metabolic Syndrome Induces Release of Smaller Extracellular Vesicles from Porcine Mesenchymal Stem Cells. *Cell Transplant*. **28**, 1271-1278, (2019).

4 Eirin, A. *et al.* Metabolic Syndrome Interferes with Packaging of Proteins within Porcine Mesenchymal Stem Cell-Derived Extracellular Vesicles. *Stem Cells Transl Med*. **8**, 430-440, (2019).

5 Coumans, F. A. W. *et al.* Methodological Guidelines to Study Extracellular Vesicles. *Circ Res*. **120**, 1632-1648, (2017).

6 Turco, A. E. *et al.* Specific renal parenchymal-derived urinary extracellular vesicles identify age-associated structural changes in living donor kidneys. *J Extracell Vesicles*. **5**, 29642, (2016).

7 Sun, I. O. *et al.* Loss of Renal Peritubular Capillaries in Hypertensive Patients Is Detectable by Urinary Endothelial Microparticle Levels. *Hypertension*. **72**, 1180-1188, (2018).

8 Sweetwyne, M. T. *et al.* The mitochondrial-targeted peptide, SS-31, improves glomerular architecture in mice of advanced age. *Kidney Int*. **91**, 1126-1145, (2017).

**
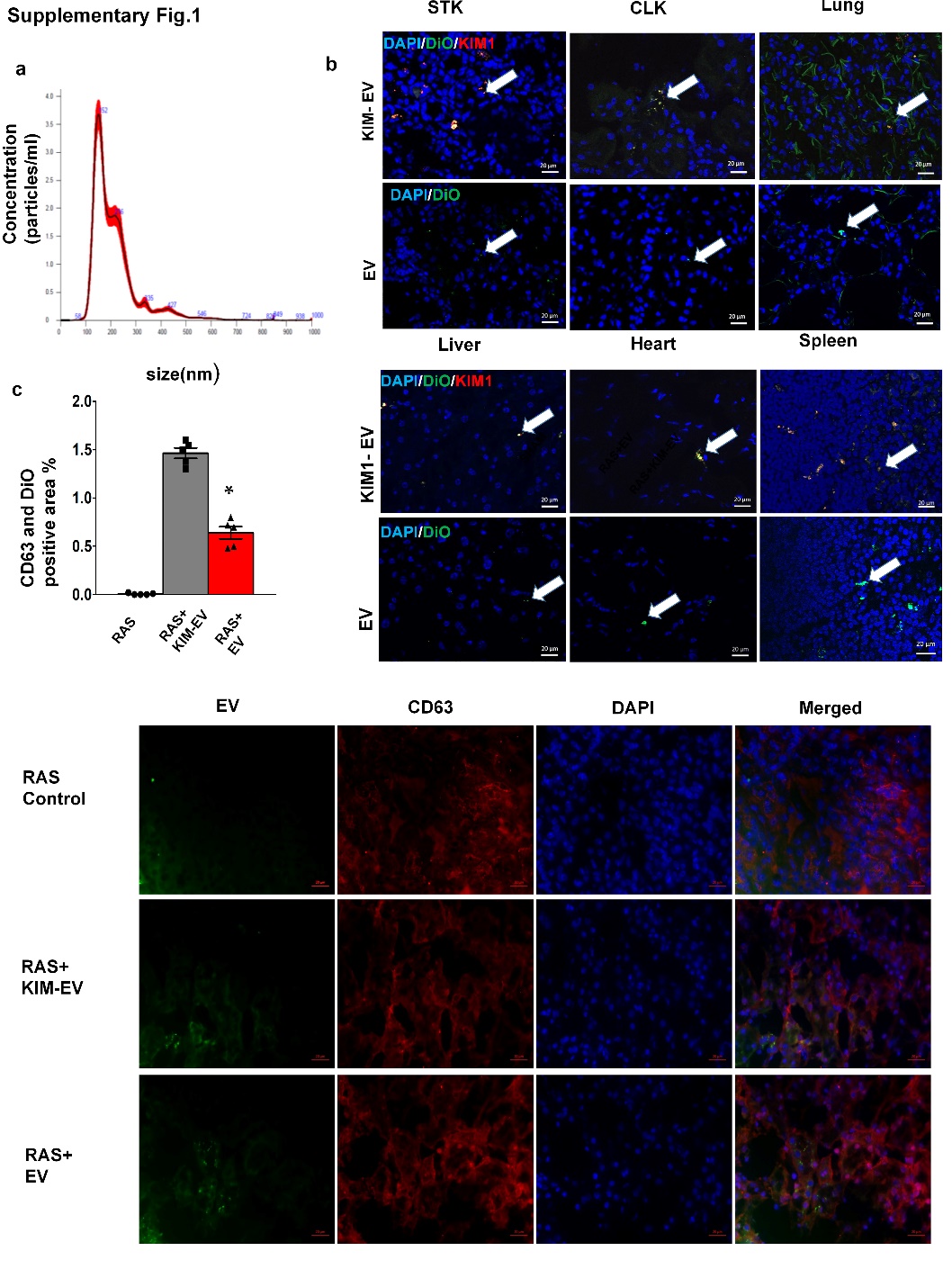
**

**Supplementary Figure 1. a.** Nano-Sight analysis of purified EVs showing their size distribution. **b.** Representative images of DiO-labeled (green) EV or DiO+APC+KIM1 (green and red) EV in the STK, CLK, lung, spleen, liver and heart. **c.** EV retention rate in kidney was calculated as percentage of double positive area per field (CD63 and DiO positive), which was higher in RAS+KIM-EV (p<0.01).

**
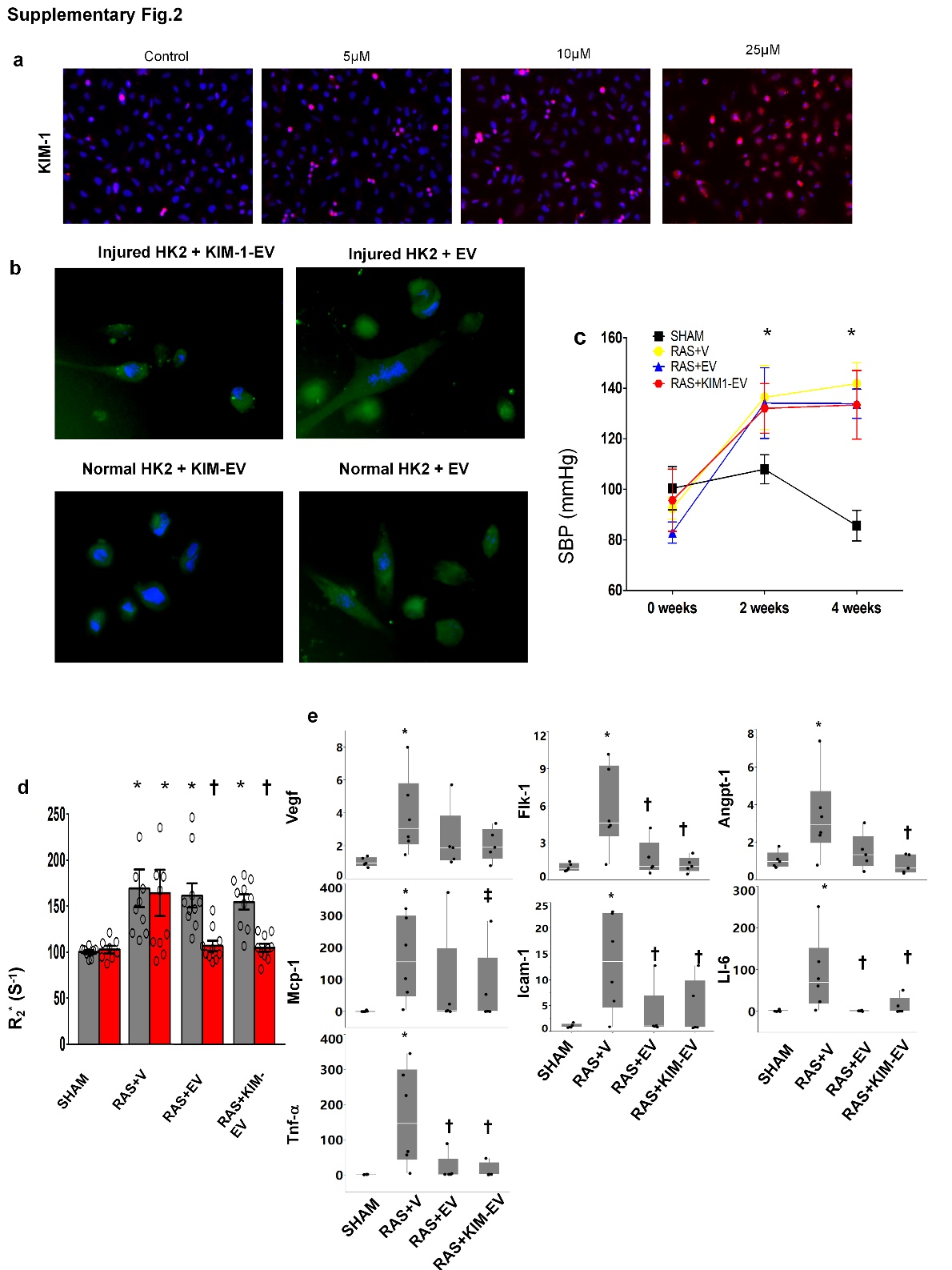
Supplementary Figure 2. a.** KIM1 expression was up-regulated with the increase of cisplatin concentration (red fluorescence). **b.** KIM-EVs or EVs (green spot) bound to injured HK2 cells. **c.** Systolic blood pressure (SBP) was measured bi-weekly in mice after RAS or sham surgery. All RAS groups showed an increase of SBP compared to Sham, which remained elevated 4 weeks after surgery (i.e., 2 weeks after EV injection) regardless of treatment. **d.** Significant STK medullary hypoxia in RAS+vehicle was alleviated by both EV and KIM‐EV, whereas STK cortical hypoxia was unchanged by either. *p<0.05 vs. Sham, † p<0.05 vs. RAS+vehicle. **e.** Renal gene expression of *Icam-1*, *Il-6, Tnf-α,Vegf, Mcp-1,*  *Flk-1,* and *Angpt1* quantified by RT-PCR (relative to GAPDH). Expression of all the tested genes were up-regulated in RAS+V compared with in Sham group. Both EV and KIM-EV decreased gene expression of *Vegf, Flk-1,* *Icam-1*, *Il-6,* and *Tnf-α* to a similar extent*. Angpt1* gene expression was decreased by native EV, but fully normalized by KIM-EV, while *Mcp-1* gene expression strongly tended to be decreased only by KIM-EV. *p<0.05 vs Sham, † p<0.05 vs RAS+vehicle, ‡ p=0.07 vs RAS+vehicle.
